# Effects of Physical Activity on College Students’ Subjective Well-Being During COVID-19

**DOI:** 10.1007/s44197-022-00062-4

**Published:** 2022-10-05

**Authors:** Shijing Yuan, Maolin You

**Affiliations:** College of Physical Education of China University of Geosciences, Wuhan, Hubei China

**Keywords:** COVID-19 pandemic, Major public health event, College students, Quality of physical activity, Subjective well-being

## Abstract

**Background:**

The COVID-19 outbreak has caused widespread psychological distress to Chinese college students. To explore the beneficial psychological effects of physical activity, this study accessed the relationship of Physical Activity (PA) and Subjective Well-being (SWB) among Chinese college students during the pandemic.

**Methods:**

A total of 1198 college students (aged between 17 and 40) from 8 universities in Wuhan, China, volunteered to finish the online questionnaire survey from February 17 to 20, 2020. General Well-Being Schedule (GWBS) was used to evaluate SWB, and Physical Activity Rating Scale-3 (PARS-3) was used to measure PA. The Mann–Whitney *U* test, *χ*^2^ test, *t* test, and analysis of variance were used to compare the differences between groups based on different data types. A multi-factor linear regression analysis was performed on the factors affecting college students' participation in physical activity during the pandemic. Differences were considered statistically significant when *p* < .05.

**Results:**

It found that: (1) The quality of physical activity during COVID-19 significantly and positively predicted subjective well-being (*B* = 2.512, *p* < .001), indicating that physical activity can effectively alleviate adverse mental health effects caused by the pandemic. (2) The pandemic has had a greater impact on the mental health of specific groups (such as seniors and rural college students). Supporting and encouraging them to participate in a certain level of sports activities can improve their subjective well-being, which is helpful for countering the pandemic’s adverse effects. (3) People should be encouraged to participate in sports at moderate or high levels.

**Conclusion:**

PA can effectively alleviate the negative psychological impact of the pandemic. In general, during major public health emergencies, people should be supported and encouraged to regularly participate in physical activities at moderate or higher levels, to improve their subjective well-being, and maintain positive anti-pandemic attitudes and behavior.

## Introduction

At the end of 2019, the sudden outbreak of the new coronavirus disease (COVID-19) pandemic has caused widespread psychological problems. Therefore, health authorities have focused on how to help the public to cope effectively with the pandemic. On January 26, 2020, new coronavirus infection of pneumonia pandemic emergency psychological crisis intervention guidelines issued by the National Health Committee emphasized the importance of addressing the psychological crisis during the pandemic. Sports are considered to be important in promoting well-being, so the State Sports General (January 30, 2020) issued the Notice on vigorously promoting the scientific fitness method, requiring various sports departments to actively advocate home fitness, by launching a simple, scientific, and effective home fitness method [1]. Therefore, guaranteeing access to national sports guidance during sudden public health emergencies (such as infectious diseases) has considerable social significance.

Subjective well-being (SWB) reflects people's subjective quality of life. It refers to the overall evaluation of one’s quality of life based on one’s own standards and includes emotional (positive and negative emotions) and cognitive (satisfaction with life) aspects [2]. SWB is a common indicator used to assess people's mental health levels. Physical activity refers to any body movement of the skeletal muscle, requiring energy expenditure, including all movements that are consumed during both leisure time and work hours [3]. As early as 1938, Jacobson found that special movement (progressive relaxation) can reduce anxiety, which means that physical activities are closely related to the emotional dimensions of subjective happiness. Existing research across different regions and countries shows that there is a positive relationship between sports activities and happiness. Although the best sports model has not yet been established, sports activities, even in small amounts, are widely recognized as being positively related to happiness [4–6]. Specifically, a meta-analysis showed that sports activities were positively related to subjective happiness, based on 980 effects, with a total effect *D* = 0.36, 95% CI [0.301, 0.420]. In an experimental study, the relationship between sports activities and subjective happiness was stronger, and organized sports activities had larger effects than free activities [7].

That sports activities can actively enhance SWB serves as a reminder that maintaining national fitness exercises during a major public health emergency can help optimize the social psychological atmosphere to form an effective overall response to the pandemic. However, the effects of a major public health event are unique, and researchers have pointed out that some people who have previously maintained exercise habits have been forced to reduce them because of the pandemic, which has had a much more negative impact on them than people who have not previously maintained exercise habits [8]. Therefore, whether sports activities still contribute to people’s subjective happiness during public health events is unclear, as are the effects. Clarifying these issues can help provide reference suggestions for utilizing people’s fitness work to ensure their mental health when major public health events emerge in the future. Considering that college students are more frequently exposed to a variety of complicated network information during the pandemic, negative emotions are highlighted [9], while at the same time, academic pressure, uncertainty about the future, lack of social support, and loneliness caused by reduced social contact can reduce their subjective happiness [10–14], The pandemic has had a more prominent effect on this group of people, so this study investigated a sample of college students.

## Materials and Methods

### Samples

The participants came from eight universities in Wuhan (Wuhan University, Huazhong University of Science and Technology, Wuhan University of Technology, China University of Geosciences [Wuhan], Huazhong Agricultural University, Hubei University, Wuhan University of Science and Technology, Wuhan University of Technology). We collected 1277 questionnaires, of which 1198 were valid, including 673 males (67.3%) and 525 females (43.8%). In the sample, 401 (33.5%) were freshmen, 516 (43.1%) sophomores, and 133 juniors (11.1%), 64 seniors (5.3%), 84 postgraduates (7.0%), all aged between 17 and 40. 626 people (52.3%) lived in cities (including counties) during the pandemic, and 572 (47.7%) in rural areas. All the participants were informed of the voluntary and confidential nature of the research, and the answer content will only be used for scientific research.

### Measures

#### General Well-Being Schedule (Chinese Version)

The revised version of the General Well-Being Schedule (GWBS) developed by Fazio [15] and adapted by Duan Jianhua [16] was used. The scale uses a total of 18 items; each item has 5–7 choices, divided into six dimensions: concerns about health, energy status, satisfaction and interest in life, happy or depressed mood, control over emotions and behavior, and degree of tension or relaxation. Questions 2, 5, 6, and 7 use a 5-point Likert-style scoring method, questions 1, 3, 4, and 8–14 use a 6-point Likert-style scoring method, and questions 15–18 use a 10-point Likert-style scoring method. Reverse scoring is used for negative questions, and a total score is calculated for each dimension. The higher the scale’s total score, the greater the subjective well-being [16]. The Cronbach's alpha of the scale in this study is 0.857, indicating that the questionnaire has good reliability.

#### Physical Activity Rating Scale-3 (Chinese Version)

We used the Physical Activity Rating Scale-3 (PARS-3) revised by Liang [17]. This scale examines the amount of exercise from three aspects: the intensity, time, and frequency of participating in physical exercise. Amount of exercise = intensity × time × frequency. Intensity and frequency are scored from 1 to 5 points; time is scored from 0 to 4 points. The highest score is 100, and the lowest is 0. Regarding the evaluation criteria for the amount of exercise: < 19 is categorized as low volume; 20–42 is categorized as medium volume; > 43 is categorized as large volume. The test–retest reliability of each PARS-3 item ranges from 0.838–0.942, so the questionnaire has good reliability.

### Procedure

From February 17–20, 2020, we ran the electronic questionnaire compiled by the questionnaire star platform, using a convenience sampling survey method. Twelve physical education teachers from the above eight universities were invited to forward the questionnaire link address (https://www.wjx.cn/jq/57644127.aspx) to the student group and ask them to answer online.

### Sample Quality Control

Researchers prepared electronic questionnaires based on the above items and demographic-related questions of the standardized scale, wrote questionnaire answer prompts, and required the participants to independently complete and truly answer the questions. After the questionnaire was collected, all questionnaires were reviewed. Among them, respondents who made obvious logical mistakes, or answered questions with a special law were eliminated.

### Statistical Analysis

Excel 2010 software was used to collect and organize data, and SPSS 25.0 was used to analyze the data. Count data are expressed by frequency and rate. Furthermore, The Mann–Whitney *U* test, *χ*^2^ test, *t* test, and analysis of variance were used to compare the differences between groups based on different data types. Moreover, a multi-factor linear regression analysis was performed on the factors affecting college students' participation in sports activities during the pandemic. Differences were considered statistically significant when *p* < 0.05.

## Results

### Quality of College Students’ Physical Activity During the Pandemic

During the pandemic, the average score of college students' sports activity quality was 15.99 ± 18.235, which means that the quality of college students' physical activities during the pandemic was low. Since the quality of physical activity is measured by a graded variable, the non-parametric Mann–Whitney *U* test (indicated by the *Z* value) and Kruskal–Wallis test (indicated by the *χ*^2^ value) were used. Significant differences were identified (Table 1), while the difference between urban and rural areas was insignificant.

**Table 1:** College students' participation in physical activities during COVID-19

Demographic variables	Sports activity quality			Test volume		*P*
	Volatility	Moderate exercise	Heavy exercise			
Gender						
Male	451 (67%)	138 (20.5%)	84 (12.5%)	*Z*	– 4.01**	0.000
Female	403 (76.8%)	91 (17.3%)	31 (5.9%)			
Age						
17–40	…	…	…	*χ*^2^	46.612**	0.000
Grade						
Freshman	303 (75.6%)	67 (16.7%)	31 (7.7%)	*χ*^2^	22.304**	0.000
Sophomore	379 (73.4%)	96 (18.6%)	41 (7.9%)			
Junior	84 (63.2%)	33 (24.8%)	16(12%)			
Senior	40 (62.5%)	15 (23.4%)	9(14.1%)			
Postgraduate (Master/PhD)	48 (57.1%)	18 (21.4%)	18 (21.4%)			
Area						
City (including county seat)	456 (72.8%)	112 (17.9%)	58 (9.3%)	*Z*	– 1.188	0.235
Rural area	398 (69.6%)	117 (20.5%)	57 (10%)			

*The significance level is 0.05

**The significance level is 0.01

### Subjective Well-Being of College Students During the Pandemic

College students' overall SWB score during the pandemic was 79.20 ± 13.22, while the national norm score was 75 for men and 71 for women, which indicates that college students displayed higher emotional well-being levels than the norm during the pandemic. Among them, the graded difference was significant (*F* = 2.684, *p* < 0.05) (Table 2). The difference between seniors and masters or doctors is the most obvious, with an average difference of 4.82 (*p* < 0.05), that between juniors and seniors was – 4.54 (*p* < 0.05), that between sophomores and seniors was – 3.46 (*p* < 0.05), and, finally, that between freshman and junior was 2.76 (*p* < 0.05) (see Table 2). However, the differences in gender, age, and urban and rural areas were not significant (Table 3).

**Table 2:** Subjective well-being of college students during COVID-19

Demographic variables	*N*	*M* ± SD	Test volume		*P*
Gender					
Male	673	78.98 ± 13.351	*t*	– 0.652	0.515
Female	525	79.48 ± 13.049			
Age					
17–40	…	…	*F*	1.532	0.076
Grade					
Freshman	401	80.34 ± 12.870	*F*	2.684*	0.030
Sophomore	516	78.66 ± 13.336			
Junior	133	77.59 ± 13.088			
Senior	64	82.13 ± 12.911			
Postgraduate (Master/PhD)	84	77.31 ± 13.994			
Area					
City (including county seat)	626	79.61 ± 12.932	*t*	1.135	0.257
Rural area	572	78.74 ± 13.518			

*The significance level is 0.05

**Table 3:** Post-test of differences in grades

Mean difference	Freshman	Sophomore	Junior	Senior	Postgraduate (Master/PhD)
Freshman	–				
Sophomore	1.677	–			
Junior	2.755*	1.078	–		
Senior	– 1.783	– 3.460*	– 4.539*	–	
Postgraduate (Master/PhD)	3.032	1.355	0.277	4.815*	–

*The significance level is 0.05

### Relationships Between the Quality of Physical Activities and the Subjective Well-Being of College Students During the Pandemic

There were significant correlations between the quality of physical activity and overall SWB and its various sub-dimensions (*P* < 0.05). Among them, the quality of physical activity was significantly negatively correlated with “health concerns” and significantly positively correlated with other dimensions (Table 4).

**Table 4:** Correlation analysis of physical activity quality and subjective well-being

	Variables	*M*	SD	1	2	3	4	5	6	7	8	9
1	Overall happiness score	79.20	13.216	1								
2	Satisfaction and interest in life	6.80	1.979	0.692^b^	1							
3	Worries about health	7.32	2.220	0.096^b^	– 0.133^b^	1						
4	Energy	19.21	4.209	0.847^b^	0.611^b^	– 0.158^b^	1					
5	Melancholy or happy mood	16.16	3.354	0.868^b^	0.548^b^	– 0.084^b^	0.721^b^	1				
6	Control of emotions and behavior	13.29	2.500	0.775^b^	0.562^b^	– 0.082^b^	0.621^b^	0.630^b^	1			
7	Relaxation and tension	16.42	3.897	0.826^b^	0.421^b^	0.120^b^	0.554^b^	0.670^b^	0.536^b^	1		
8	Sports activity quality	1.38	0.655	0.105^b^	0.109^b^	– 0.062^a^	0.140^b^	0.100^b^	0.059^a^	0.061^a^	1	

^a^The significance level is 0.05

^b^The significance level is 0.01

We performed a multi-factor linear regression analysis (Table 5), taking the college students’ overall SWB scores and sub-dimensions scores as dependent variables, and demographic variables and physical activity quality as predictor variables (Table 4). The results showed that each model was significant (*p* < 0.05).

**Table 5:** Determination of Variables

Variables	Assignment situation
Dependent variable	
Overall well-being and its six sub-dimensions	Actual score
Independent variable	
Gender	Male = 1; female = 2
Grade	Freshman = 1; sophomore = 2; junior = 3; senior = 4; Master and doctoral students = 5
Age	Actual age
Area	City (including county seat) = 1; Rural area = 2
Quality of physical activity	Fluctuation = 1; Moderate exercise = 2; Heavy exercise = 3

We established seven regression models for analysis (Table 6), taking overall SWB and its dimensions as the dependent variables of each model and physical activity quality as the independent variable. The quality of college students' physical activity during the pandemic was related to overall SWB. All of its dimensions had significant effects (*p* < 0.05), which is consistent with the results of the correlation analysis. After incorporating demographic variables into the regression model, the factors that significantly predicted college students’ overall SWB were age (*p* < 0.01) and quality of physical activity (*p* < 0.001). The following results were found for the sub-dimensions of SWB:

1. Only the quality of physical activity significantly predicted “satisfaction and interest in life” (*p* < 0.001), “energy” (*p* < 0.001), and “depressed or happy mood” (*p* < 0.01).
2. Only age had a significant effect on “concerns about health” (*p* < 0.01).
3. Both quality of physical activity and age significantly predicted “overall well-being” (*p* < 0.01) and “relaxation and tension.” Differences between urban and rural areas significantly predicted “control of emotion and behavior” (*p* < 0.01).

**Table 6:** Multi-factor regression analysis of physical activities affecting college students' subjective well-being during COVID-19

Model number	Dependent variable	Independent variable						*F*	*R*^2^
		*B*							
		Constant	Gender	Age	Grade	Area	Sports activity quality		
1	Overall happiness score	86.886**	–	– 0.552**	–	–	2.512**	12.012**	0.020
2	Satisfaction and interest in life	6.343**	–	–	–	–	0.330**	14.471**	0.012
3	Worries about health	9.727**	0.191	0.126**	0.047	–	– 0.155	5.125**	0.017
4	energy	17.968**	–	–	–	–	0.899**	23.831**	0.020
5	Melancholy or happy mood	15.447**	–	–	–	–	0.514**	12.148**	0.010
6	Control of emotions and behavior	13.518**	–	–	–	– 0.375**	0.233*	5.463**	0.009
7	Relaxation and tension	19.792**	–	– 0.197*	– 0.027	–	0.478**	6.681**	0.017

*The significance level is 0.05

**The significance level is 0.01

### Comparative Analysis of Subjective Well-Being of College Students at Different Levels of Physical Activity Quality During the Pandemic

The analysis results show that the SWB score of low exercise subjects is 78.40 ± 13.321, that of moderately active subjects is 80.41 ± 12.288, and that of high-volume subjects is 82.71 ± 13.570. The result of the test of homogeneity of variance was homogeneous (*p* > 0.05), therefore, analysis of variance could be performed. The results show that there were significant differences in the SWB scores of college students under different levels of physical activity, *F*(2, 1195) = 6.662, *p* < 0.01, (*η*^2^ = 0.011), and the SWB score of low exercise subjects is significantly lower than that of moderately active subjects (*p* < . 05) and high-volume subjects (*p* < . 001), but there is no significant difference between the moderately active subjects and high-volume subjects, which means that we should encourage the college students to participate in medium or high-level sports activities. The test results of the homogeneity of variance test of the six sub-dimensions of subjective well-being were all non-significant (*p* > 0.05), meaning the analyses of variance could all be performed. Further analysis showed significant differences in the “overall happiness score,” “satisfaction and interest in life,” “energy,” and “depressed or pleasant mood” of college students within different physical activity quality levels (*p* < 0.05). Moreover, this difference was not significant for “worries about health,” “control of emotions and behaviors,” and “relaxation and tension”.

## Discussion

### Sports are an Important Measure to Support College Students to Maintain an Active Attitude When Fighting the Pandemic

This study shows that physical exercise during the COVID-19 pandemic predicted college students’ positive emotions and subjective well-being. College students with high-quality physical activity (large amounts of exercise) had stronger subjective well-being, higher satisfaction with life, more energy, more pleasant mood, better control of emotions and behaviors, and felt relaxed more easily. This is consistent with many previous research results [18–21]. Sports may improve the mechanism of physical activity and help people obtain a good participation experience, making people feel physically relaxed, happy, and full of energy, and enhance self-efficacy and life satisfaction. At the same time, the stress-buffering effect of physical exercise can help alleviate the insecurity, reducing adverse effects on mental health and well-being caused by the new COVID-19 pandemic [22, 23]. Furthermore, participating in sports can provide more social support, which has a positive protective and enhancing effect on mental health and well-being [24]. Therefore, sports can be an important measure for college students to protect their physical and mental health during major public health incidents. Educational departments and universities at all levels should actively take measures (such as online physical education classes, online sports competitions, and after-school physical education assignments, etc.) to support and encourage college students to participate in physical exercises. Doing so will help people maintain and enhance their subjective well-being, establish a positive anti-pandemic mentality, and help them effectively fight the pandemic, live normally, and study.

### Senior College Students Should Actively Participate in Physical Exercise During the Pandemic

During the pandemic, senior college students faced more serious threats to their SWB, which is consistent with the result of Liu’s research [13]. Graduation and employment are urgent tasks that senior college students face. The resulting stress will affect their mental health and cause higher anxiety levels. Therefore, senior college students have lower SWB. However, senior college students have fewer academic tasks and more time at their disposal during the pandemic. This provides opportunities for them to participate in physical exercise. With aging, their health awareness increases, and they pay more attention to the important value of physical exercise to maintain a healthy lifestyle. Therefore, during the pandemic period, we should focus on and support senior college students in participating in physical exercise and adopting practical and effective measures to help them improve their SWB through physical exercise. This could help them to better cope with the pressure of graduation and employment, avoid psychological problems.

### Pay Attention to Rural College Students’ Physical Exercise During the Pandemic

The SWB of rural college students during the pandemic also needs attention. This study showed that rural college students’ ability to control emotions and behaviors was significantly lower than that of urban college students during the pandemic. During the pandemic, they may have limited access to life, social, and learning support, resulting in emotional and behavioral problems. The ability to control emotions is significantly lower than that of urban college students, negatively affecting their sense of control over emotions and behaviors. However, this difference may reflect a different cultural background, or even seasonal differences [25]. Therefore, we can reasonably use the rural landscape environment to encourage them to participate in outdoor sports activities (such as mountain climbing and hiking) and provide appropriate exercise guidance (such as training videos, graphics, and texts) and sports equipment (such as dumbbells, skipping ropes, and ball games) during the pandemic. This will help them maintain positive physical and mental health and well-being during the pandemic, which is of great value in consolidating pandemic prevention work in rural areas and maintaining safety and stability in colleges and universities.

## Conclusion

Major public health incidents have caused serious threats to people’s well-being and have especially had an impact on senior and rural college students. Sports could help improve their subjective happiness during the pandemic. Based on the findings of the study, the following recommendations are made: (1) During major public health incidents, we should support and encourage people to participate in sports activities, to a moderate or high degree, to maintain and improve their subjective happiness and support pandemic prevention efforts; (2) The pandemic poses a greater threat to the mental health of residents who are under greater pressure for survival and development, especially rural residents, so more attention should be paid to this specific part of the population to support and encourage them to improve the quality of their sports activities; (3) Participating in medium or high-level sports activities could also effectively alleviate the negative psychological effects of the pandemic.

## Data Availability

The data and material used in this study are available, we consent to upload if it is necessary.

## Abbreviations

PA: Physical activity (PA) reflects people’s participation in sports and exercise
SWB: Subjective well-being (SWB) reflects people's subjective quality of life
GWBS: General Well-Being Schedule (GWBS) is a tool used to explore people’s SWB
PARS-3: Physical Activity Rating Scale-3(PARS-3) used to examine the quality of physical activity
